# The G2A Receptor Deficiency Aggravates Atherosclerosis in Rats by Regulating Macrophages and Lipid Metabolism

**DOI:** 10.3389/fphys.2021.659211

**Published:** 2021-07-26

**Authors:** Xueqin Cui, Roumei Xing, Yue Tian, Man Wang, Yue Sun, Yongqian Xu, Yiqing Yang, Yongliang Zhao, Ling Xie, Yufang Xiao, Dali Li, Biao Zheng, Mingyao Liu, Huaqing Chen

**Affiliations:** ^1^Shanghai Key Laboratory of Regulatory Biology, School of Life Sciences, Institute of Biomedical Sciences, East China Normal University, Shanghai, China; ^2^Department of Pathology & Immunology, Baylor College of Medicine, Houston, TX, United States

**Keywords:** G2A, atherosclerosis, LDLR deficient rat, lipid disorder, macrophage

## Abstract

The orphan G protein-coupled receptor G2A has been linked to atherosclerosis development. However, available data from mouse models are controversial. Rat G2A receptor bears more similarities with its human homolog. We proposed that the atherosclerosis model established from *Ldlr*^–/–^ rat, which has been reported to share more similar phenotypes with the human disease, may help to further understand this lipid receptor. G2A deletion was found markedly aggravated in the lipid disorder in the rat model, which has not been reported in mouse studies. Examination of aortas revealed exacerbated atherosclerotic plaques in G2A deficient rats, together with increased oxidative stress and macrophage accumulation. In addition, consistently promoted migration and apoptosis were noticed in G2A deficient macrophages, even in macrophages from G2A single knockout rats. Further analysis found significantly declined phosphorylation of PI3 kinase (PI3K) and AKT, together with reduced downstream genes Bcl2 and Bcl-xl, suggesting possible involvement of PI3K/AKT pathway in G2A regulation to macrophage apoptosis. These data indicate that G2A modulates atherosclerosis by regulating lipid metabolism and macrophage migration and apoptosis. Our study provides a new understanding of the role of G2A in atherosclerosis, supporting it as a potential therapeutic target.

## Introduction

As a chronic disease affecting arteries, atherosclerosis is characterized by lumen narrowing progressively developed from gradual expansion of lesions, which may eventually lead to myocardial infarction or stroke. It has been largely accepted that both lipid disorder and chronic inflammation are involved in atherosclerosis. The complicated interplay between inflammation and lipid metabolism leads to immune cell infiltration and aggravated lesions in the arterial wall (Moore and Tabas, 2011; Shalhoub et al., 2011; Fogelstrand and Boren, 2012; van Diepen et al., 2013).

As the predominant immune cell type, macrophages around lesions are thought to originate from bone marrow; in which hematopoietic stem cells give rise to circulating monocytes (Tabas and Lichtman, 2017). It is believed that macrophage accumulation in the intima and subintima of arteries is vital for the formation of obstructive atherosclerotic plaques. The number of infiltrated macrophages and their location at plaque rupture-sensitive sites is related to plaque vulnerability (Davies et al., 1993). As part of their general homeostatic scavenging function, macrophages could consume toxic lipids, such as oxidized low-density lipoproteins (oxLDL) (Acton et al., 1996). Increased oxLDL is associated with the formation of lipid-laden macrophages-foam cells in lesional sites, which is critical in atherosclerosis development (Kadl et al., 2010; Chinetti-Gbaguidi and Staels, 2011). The pathological mechanism of atherosclerosis suggest that, although lipid-lowering therapy represents the mainstream strategy at the moment, the possible combination of restoring imbalanced immunity could become a promising new goal for combating the disease.

The orphan G protein-coupled receptor G2A (GPR132) got its name because it induces G2 cell cycle arrest (Weng et al., 1998). It is activated by lysophospholipids and free fatty acids including lysophosphatidylcholine (LPC) and 9-hydroxyoctadecadienoic acid (9-HODE), which related to oxLDL (Obinata et al., 2005; Yang et al., 2005; Frasch et al., 2007; Foster et al., 2019). G2A is highly expressed in immune tissues, also on immune cells including macrophages and lymphocytes. It is also expressed in endothelium (Rikitake et al., 2002; Parks et al., 2005). In addition to modifying cell cycle and oncogenesis, research data about G2A mainly concentrated on its function in immunity and inflammation (Peter et al., 2008; Bolick et al., 2009; Frasch et al., 2011; Qin et al., 2014), though a few suggested its involvement in hepatic lipid metabolism (Johnson et al., 2008).

In humans or mice, G2A was found predominantly expressed in monocytes/macrophages within plaques, suggesting its involvement in atherosclerosis initiation or progression (Rikitake et al., 2002). Quite a few studies had been conducted in mouse atherosclerotic models and found that G2A showed regulatory effects on atherosclerosis development through alterations of macrophages, endothelium, or other factors. However, both proatherogenic and atheroprotective actions of G2A had been described (Parks et al., 2005, 2006, 2009; Bolick et al., 2009). Some stated that G2A deletion led to macrophage activation and less apoptosis which was associated with more macrophages in aortas and increased atherosclerosis (Bolick et al., 2009). Others found G2A deficiency attenuated atherosclerosis owing to HDL increase, independent of macrophages (Parks et al., 2006, 2009). By bone marrow transplantation, bone marrow-derived cells were found both participated and not participated in G2A regulating atherosclerosis (Bolick et al., 2009; Parks et al., 2009). The difference reported in these articles was not related to background variation since they used the same mouse strain. As for G2A’s role on blood lipid profiles, studies from mice usually described no significant effect, but some reported HDL modulation in *Ldlr*^–/–^ model after prolonged diet intervention (Parks et al., 2005, 2009; Bolick et al., 2009). The above information indicates that mouse study alone is not sufficient to elucidate G2A’s impact on atherosclerosis. New animal systems are hence required to help to clarify G2A mediated effects on different aspects of atherosclerosis including macrophages and lipid metabolism.

Rats have been used extensively as human disease models with their advantages. When we looked at the amino acid sequence of rat G2A, it shares more similarity with human G2A than the mouse homolog does: 60 vs. 21% similarity in the N-terminal extracellular domain and 73 vs. 41% in C-terminal cytoplasmic tail (Obinata and Izumi, 2009). Moreover, we have developed genetically modified rat models of atherosclerosis and found that they share more closely related human phenotype than mice in certain aspects, including heavier plaques in males (Zhao et al., 2018). Thus in this study, we assessed the role of G2A in atherosclerosis using the rat model to get a better understanding of the receptor. Our results showed that G2A deletion greatly aggravated dyslipidemia, potentiated macrophage migration and apoptosis in LDLR deficient rats, and eventually led to more macrophage accumulation and lesion development in the aortic wall.

## Materials and Methods

### Animals

The total *Ldlr* gene knockout (*Ldlr*^–/–^) rat was previously established by the CRISPR/Cas9 system and maintained in our laboratory (Zhao et al., 2018). In the current study, a heritable total *G2a* knockout (*G2a*^–/–^) rat was generated in the same way. Briefly, two sgRNAs targeting the exon 3 of *G2a*, CTGCCACACGTTGTCCTACGAGG, and CGGCGAGCACGTTTCTCTGTAGG, were constructed by overlapping PCR. The transcribed sgRNA and Cas9 mRNA were purified and a mixture of them was microinjected into zygotes of Sprague Dawley rats (SLAC Laboratory Animal Co., Ltd., Shanghai, China). Three pups (founder 1–3) were born after 50 injected zygotes were transferred to 1 pseudopregnant female rat. All three pups carried *G2a* deletion. Sequence analysis revealed that these three founders had frameshift mutations (Supplementary Figures 1A,B). In the end, founder one was chosen and a stable colony of *G2a*^–/–^ rat was established, which carried 199-bp deletion from No. 137654370bp to 137654379bp and No. 137654088bp to 137654276bp in the genome (NC_005105.4), and resulted in a termination codon TAA and deletion of 346 amino acids. Rat tail clips were used for genotyping as usual (Supplementary Figure 1C). PCR primers for identifying *G2a* mutation were 5′- CCTCATCTTGTCAGGGTC -3′ (sense) and 5′- CCGCAGGTAGTAGTAGCC -3′ (antisense). Homozygous *G2a*^–/–^ rats showed a markedly reduced *G2a* expression in related tissues (Supplementary Figure 1D).

Genetically matched *Ldlr*^–/–^ and *Ldlr*^–/–^*G2a*^–/–^ rats were derived from cross-breeding *G2a*^–/–^ and *Ldlr*^–/–^ rats. Both male and female animals were maintained and monitored as described previously, in a SPF facility on 12 h light/12 h dark cycles. All rats were supplied water and a normal diet freely accessible. Animals were transferred to Western Diet at 8 week-old to induce atherosclerosis (Zhao et al., 2018). All animal procedures and techniques were approved by the Animal Ethics Committee of East China Normal University with a permit number (R20170202). A mix of male and female rats was used in this study. Most animal experiments were originally conducted by dividing male and female rats. As both genders displayed similar results, they were combined. Representative images of histological analysis and immunoblotting were from male rats. A scheme depicting the time protocol of rat treatments and time points of measuring various parameters was provided in Supplementary Figure 2.

### Bone Marrow-Derived Macrophages and Foam Cell Induction

Bone Marrow-Derived Macrophages (BMDMs) were induced from bone marrow derived cells of 8 or 36 week-old animals as previously described (Weischenfeldt and Porse, 2008; Muschter et al., 2015), and about 90% of the resulted cells were CD68 positive. For foam cell induction, macrophages were incubated with 10 μg/ml oxLDL for 24–48 h. In some experiments, macrophages were stimulated with 100 ng/ml LPS (Sigma) or 20 ng/ml IL-4 (PeproTech) and their expression of inflammatory or anti-inflammatory cytokines were then evaluated by quantitative real-time PCR.

### Cell Migration Assay

Migration assay was performed using the transwell chamber (Corning Falcon) in a 24-well cell culture plate. Cell suspensions containing 1 × 10^5^ cells in 500 μl serum-free DMEM medium with 10 μg/ml oxLDL were placed in the top chamber. The lower chamber was filled with 500 μl complete DMEM medium containing 10 μg/ml oxLDL and 10% FBS. After 36 h incubation in a 37°C, 5% CO_2_ incubator, migrated cells were fixed with 4% paraformaldehyde, washed then stained with 0.1% crystal violet for 30 min. Images were captured through an inverted microscope (Olympus), and cells counted with the ImageJ software.

### ROS Detection in HUVEC Cells

The primary human umbilical vein endothelial cell (HUVEC) was from Dr. Xinli Wang (Baylor College of Medicine, Houston, TX), and cultured as described previously (Wu et al., 2016). Expression of G2A in the cells was knocked down by siRNA (about 60% knockdown assessed by real-time PCR). ROS was detected by means of an oxidation-sensitive fluorescent probe (DCFH-DA) (ROS Assay Kit, S0033S, Beyotime Biotechnology, China). After treated with 20 μg/ml oxLDL for 36 h, cells were washed then incubated with 10 μmol/L DCFH-DA according to the manufacturer’s instructions. The dichlorofluorescein (DCF) fluorescence was detected by FlexStation 3 (Molecular Devices) at an excitation wavelength of 488 nm and emission wavelength of 525 nm. The oxidative stress marker, malondialdehyde (MDA) in serum was conducted by a plate reader assay, based on reaction between MDA and thiobarbituric acid, as described in Lipid Peroxidation MDA assay kit (S0131S, Beyotime Biotechnology, China). OD535nm was detected by Cytation 5 imaging reader (BioTek, United States).

### Detection of Serum Lipids

At 8, 24, and 36 week-old, rats were fastened overnight. Blood samples were collected under anesthesia through intraperitoneal injection of 1.25% (m/v) avertin (Sigma-Aldrich). Serum was obtained by centrifugation at 3,000 rpm for 15 min at 4°C, and then kept at –80°C until analysis. Lipids including total cholesterol (TC), triglyceride (TG), low-density lipoprotein cholesterol (LDL-c) and high-density lipoprotein cholesterol (HDL-c) were analyzed using AU680 Automatic Biochemistry Analyzer (Beckman Coulter, United States). For atherosclerosis risk prediction, the atherosclerotic index was calculated as (TC- HDL-c)/HDL-c (Zhao et al., 2018; Zhang et al., 2020). Levels of oxLDL, free fatty acid, insulin and leptin were determined by respective ELISA assays from Shanghai Hengyuan Biological Technology Co., Ltd. (Shanghai, China).

### Quantitative Real-Time PCR Analysis

Total RNA was extracted from cells or tissues using Trizol (Invitrogen). Reverse transcription was performed with the cDNA Prime Script Reverse Transcription kit (Takara) according to the manufacturer’s instructions. Real-time PCR was performed in triplicate by SYBR Green PCR Master Mix (Takara) on a MX3005p system (Stratagene, United States). 500 ng RNA was used for the reverse transcription, and 500 ng cDNA for real-time PCR. The expression of target genes was normalized to the house keeping gene β-actin.

### Histological Analysis

The histological analysis was performed as previously published (Zhao et al., 2018). Briefly, rats were sacrificed at 36 or 54 weeks old by CO_2_ asphyxiation under avertin anesthesia. Whole aortas were excised and adipose tissue and adhesion tissue around the arteries were removed. Aortas were fixed in 4% paraformaldehyde at 4°C overnight. The fixed tissues were paraffin-embedded, and sections were prepared using a paraffin slicer (Leica) then subjected to hematoxylin-eosin (H&E) staining. The *en face* aortas or frozen sections were subjected to Oil red O staining, Masson staining or immunohistochemical staining. Stained sections were photographed by an inverted microscope (Leica) or Caseviewer2.0 (3D HISTECH), and the resulted images were analyzed by Image-Pro^®^ Plus version 6.0 software.

### Immunoblotting

Ice-cold SDS lysis buffer (0.08 mM Tris–HCl, 10 SDS, 0.25% bromophenol blue, 50% glycerol and 25% β-mercaptoethanol) was used to collect proteins from cells. Heat denatured proteins were equally loaded and fractionated by 12% SDS-PAGE, then transferred to a nitrocellulose membrane (Schleicher and Schuell MicroScience). According to the target protein, membranes were incubated with the following respective primary antibodies: rabbit anti-PI3K (1:1,000 dilution, Cat. No. 4257), rabbit anti-phospho-PI3K (1:1,000, Cat. No. 4228), rabbit anti-AKT (1:1,000, Cat. No. 9272), rabbit anti-phospho-AKT (1:2,000, Cat. No. 4060), and rabbit anti-Bcl-xL (1:1,000, Cat. No. 2764) from Cell Signaling Technology (CST), rabbit anti-Bcl-2 (1:1,000, sc-56015) from Santa Cruz and mouse anti-β-actin (1:1,000, A5441) from Sigma. Secondary antibodies were donkey anti-mouse or donkey anti-rabbit (1:2,000) from Thermo-Invitrogen. Detection of positive bands were conducted using fluorescence labeled secondary antibodies IRDye^®^ 800CW goat anti-mouse IgG (Cat. No. 926-32210) and IRDye^®^ 800CW goat anti-rabbit IgG (Cat. No. 926-32211) from Li-COR. Images of the blots were analyzed on the Odyssey Infra-Red Imaging System (Li-COR).

### Statistical Analysis

All data are expressed as mean ± SD. Statistical analysis was performed by Graphpad prism 6 software. Differences between two groups were determined by an unpaired Student’s *t*-test. For multiple comparisons, two-way ANOVA with Tukey’s multiple comparison test was used. Values of *p* < 0.05 were considered statistically significant.

## Results

### G2A Single Deficiency Did Not Alter Lipid Metabolism in WT Rats but Promoted Macrophage Migration and Apoptosis

To evaluate its possible involvement in atherosclerosis, G2A expression in atherosclerosis related tissues and cells were first analyzed in WT rats. Similar to mice G2A, rat G2A was found highly expressed in immune organs including thymus and spleen, and widely expressed in immune cells, such as lymphocytes and different subpopulations of macrophage, with the bone marrow cells showed the highest expression. There was also some G2A expression in the visceral adipose tissue (VAT) and aorta (Supplementary Figure 3).

**FIGURE 1:**
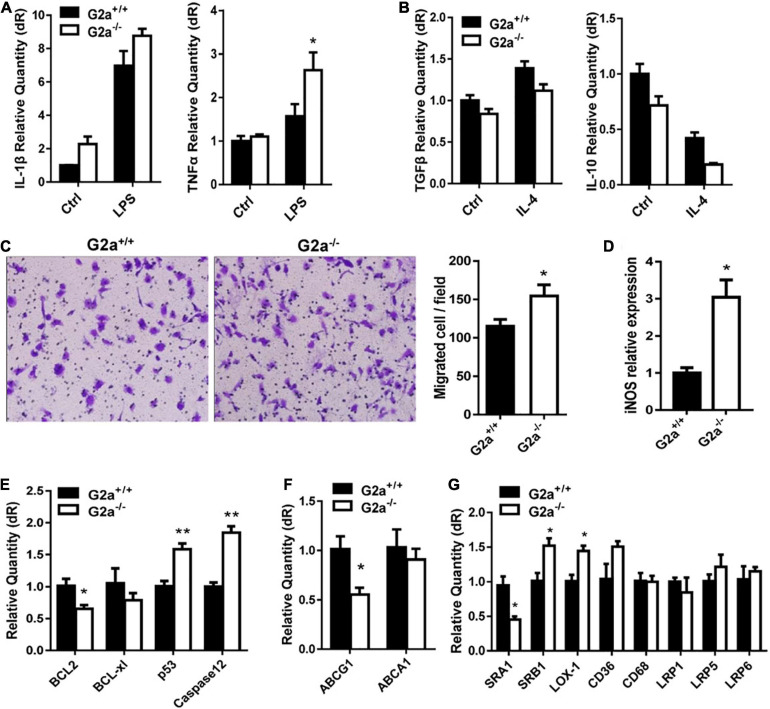
Effect of G2A single knockout on WT rats. Real-time analysis of **(A)** inflammatory cytokines expressed by LPS stimulated macrophages, and **(B)** anti-inflammatory cytokines by IL-4 stimulated macrophages from 8 week-old WT and *G2a*^–/–^ rats. **(C)** Transwell migration assay for 10 μg/ml ox-LDL stimulated macrophages. **(D)** The mRNA level of iNOS in arteries from WT and *G2a*^–/–^ rats. **(E–G)** Real-time PCR analysis of indicated genes in macrophages induced for 24 h by ox-LDL. Data are expressed as mean ± SD. *n* = 3–9. *p < 0.05, **p < 0.01 vs. WT littermates (Student’s *t*-test).

Next, phenotypes associated with atherosclerosis development including lipid metabolism, oxidative stress and macrophages were compared between *G2a*^–/–^ and WT rats. Serum lipid profile analysis did not show a significant difference in the two genotypes in either normal diet or Western diet-fed groups (Supplementary Figure 4). Neither was an obvious change discovered in cytokines secreted by macrophages, except a minor increase in the inflammatory cytokine TNFα by LPS stimulated *G2a*^–/–^ macrophages (Figures 1A,B). However, consistent enhancement was detected in macrophage migration. As shown in Figure 1C, bone marrow derived macrophages from 8 week-old *G2a*^–/–^ rats displayed enhanced migration when induced by ox-LDL. Moreover, the reactive oxygen species (ROS) producing enzyme nitric oxide synthase 2 (iNOS) was markedly increased in arteries of G2A deficient rats (Figure 1D), indicating oxidative stress and cell injury. The expression of apoptotic genes was significantly enhanced while survival genes were reduced in *G2a*^–/–^ macrophages (Figure 1E).

We further analyzed the effects of G2A deficiency on ATP binding cassette transporters and lipid scavenger receptors. ABCA1 did not show significant change, but a decreased ABCG1 mRNA level was noticed in *G2a*^–/–^ macrophages (Figure 1F). No significant difference in LDLR-related proteins (LRP1, LRP5, and LRP6) was detected. There were some changes in the expression of lipid scavenger receptors (SRs), though, with increases in SRB1 and lectin-like oxidized LDL receptor-1 (LOX-1) but a reduction in SRA1 (Figure 1G). Taken together, G2A knockout in WT rats did not affect lipid metabolism but led to arterial oxidative stress as well as enhancement in macrophage migration and apoptosis.

### G2A Deficiency Exacerbated Lipid Disorder in the *Ldlr*^–/–^ Atherosclerotic Model

**FIGURE 2:**
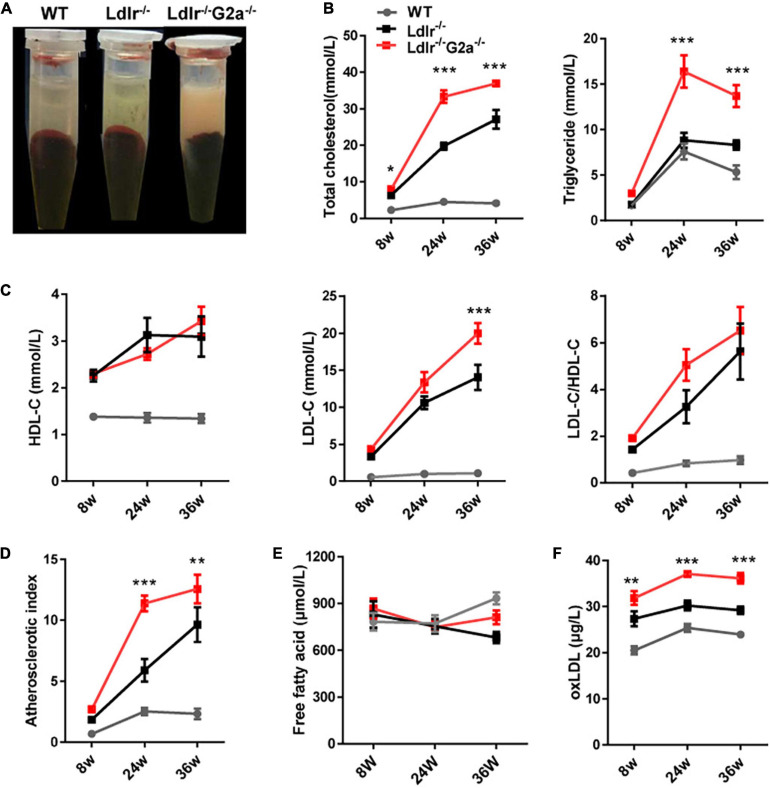
Lipid profile of WT, *Ldlr*^–/–^ and *Ldlr*^–/–^ *G2a*^–/–^ rats. **(A)** Typical appearance of sera from the three genotypes. **(B)** Total cholesterol (TC) and triglyceride (TG) level. **(C)** High density lipoprotein cholesterol (HDL-c), Low-density lipoprotein cholesterol (LDL-c), and LDL-c/HDL-c ratio. **(D)** The atherosclerosis predictor atherosclerotic index, calculated as (TC- HDL-c)/HDL-c. **(E)** Serum level of free fatty acid. **(F)** Oxidized low-density lipoprotein (oxLDL) level. Data are shown as mean ± SD and combined from male and female rats (they showed a similar pattern). *n* = 10–15. *p < 0.05, **p < 0.01, ***p < 0.001 vs. *Ldlr*^–/–^ rats (two-way ANOVA with Tukey’s multiple comparisons test).

Although G2A deficiency did not alter lipid profiles in WT rats, an apparent difference was observed between sera from 24 week-old *Ldlr* ^–/–^ and *Ldlr*^–/–^*G2a*^–/–^ rats with the former clearer and the latter more turbid (Figure 2A). Not surprisingly, greatly augmented TC and TG were detected in *Ldlr*^–/–^*G2a*^–/–^ rats. A significant increase for TC even began at 8 week-old, before Western diet induction (Figure 2B). The HDL-c level did not show significant change, but a marked elevation of LDL-c was detected at 36 week-old, along with a trend of increase in LDL/HDL ratio (Figure 2C). Interestingly, a greatly up-regulated atherosclerotic index was manifested in 24 and 36 week-old *Ldlr*^–/–^*G2a*^–/–^ rats as compared to respective *Ldlr*^–/–^ controls (Figure 2D), predicting higher atherosclerosis risk. No significant alteration was noticed in free fatty acids, but a very consistent increase was found in the serum level of oxLDL, even at 8 weeks old before the Western diet intervention (Figures 2E,F). To analyze whether the aggravated lipid disorder had anything to do with food intake and body weight, we checked the two indexes, as well as water intake, leptin, and insulin. Only water intake in male *Ldlr*^–/–^*G2a*^–/–^ displayed a moderate enhancement as compared to the male *Ldlr*^–/–^ counterpart (Supplementary Figure 5). No significant difference in these indexes was detected between female rats of the two genotypes (data not shown). Therefore, G2A deficiency severely aggravated lipid disorder in *Ldlr*^–/–^ rats which had no apparent relation to food intake or body weight.

### G2A Deletion Aggravated Atherosclerosis in the *Ldlr*^–/–^ Rat Model

**FIGURE 3:**
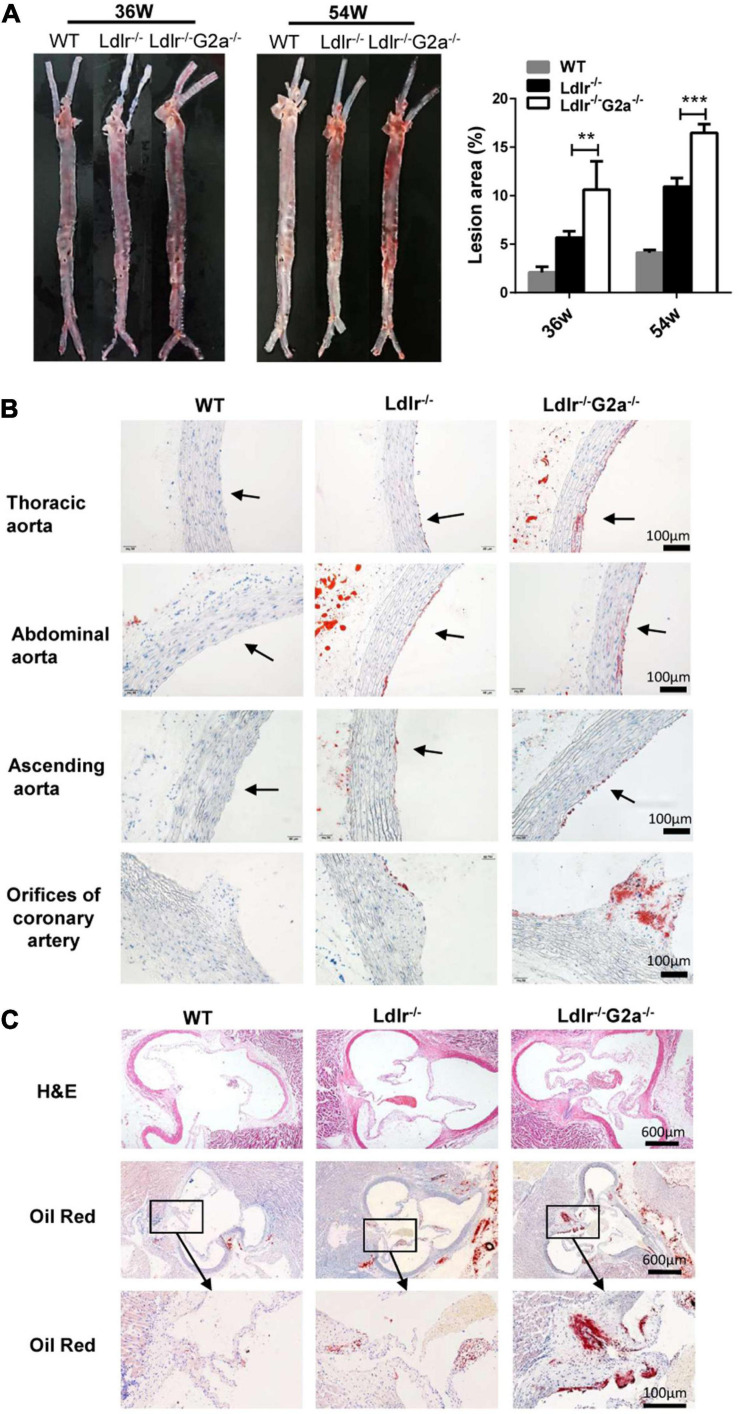
Representative images and lesion quantification of atherosclerosis in the Western diet-fed rats. **(A)** Oil Red O staining of *en face* aortas, and quantification for their lesion area (% total aorta) in 36 and 54 week-old male rats. **(B)** Oil Red O staining of aortic segments in 36 week-old rats. **(C)** H&E and Oil Red O staining of aortic sinuses from different genotypes at 36 weeks of age. Data are shown as mean ± SD (*n* = 6). **p < 0.01 and ***p < 0.001 vs. *Ldlr*^–/–^ rats (two-way ANOVA with Tukey’s multiple comparisons test).

After finding the exacerbated dyslipidemia in *Ldlr*^–/–^*G2a*^–/–^ rats, we next examined the direct effect of G2A deficiency on atherosclerosis development. Not surprisingly, male and female Western diet-fed *Ldlr*^–/–^*G2a*^–/–^ rats at 36 and 54 weeks of age showed significantly more aortic plaques by *en face* analysis, as compared to *Ldlr*^–/–^ rats. Representative *en face* aortic images were presented in Figure 3A and lesion areas accounted for 7.2–15.4% of total aortic intima in 36 week-old and 15.3–17.3% in 54 week-old *Ldlr*^–/–^*G2a*^–/–^ rats, average 1.9 and 1.5-fold increase of lesion area in respective *Ldlr*^–/–^ counterparts. Besides, histological analysis showed more lipid deposition in different segments of the *Ldlr*^–/–^*G2a*^–/–^ aorta, especially in the aortic sinus and coronary arterial orifice (Figures 3B,C). To analyze whether G2A deficiency affected other tissues, we examined the liver, spleen, heart, kidney, visceral adipose tissue (VAT), and lung. No significant change was discovered in their relative tissue weight (data not shown). However, there was more lipid deposit in the *Ldlr*^–/–^*G2a*^–/–^ liver without significant change in aspartate aminotransferase (AST) or alanine aminotransferase (ALT) levels. A similar result was noticed in the kidney of *Ldlr*^–/–^*G2a*^–/–^ rats, with more lipid deposits but no significant difference in uric acid or creatinine level. Moreover, no apparent histological change was found in the liver or kidney (Supplementary Figure 6). The collective data means G2A deficiency in *Ldlr*^–/–^ rats led to increased atherosclerosis development, and enhanced lipid deposition in the liver and kidney.

### Increased Macrophage Recruitment and Vascular Fibrosis in Aortas of G2A Deficient *Ldlr*^–/–^ Rats

**FIGURE 4:**
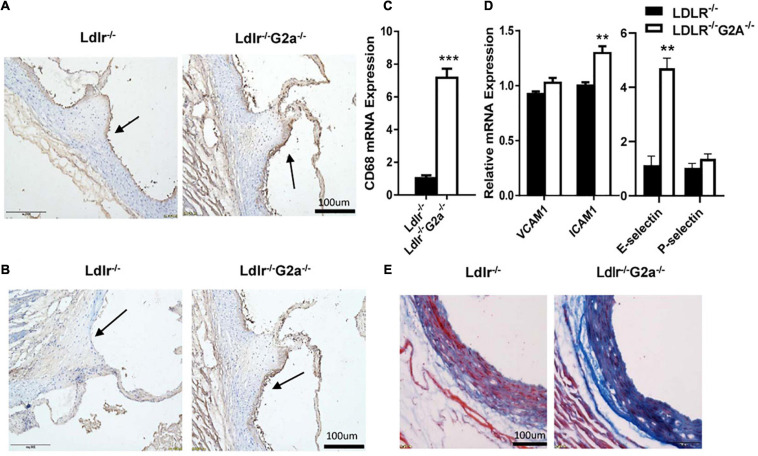
Lesional macrophage accumulation and vascular fibrosis in 36 week-old rats. **(A)** Macrophages in aortic sinus of the two indicated genotypes, identified by immunohistochemical staining with anti-CD68 antibody (Bio-Rad AbD Sereotec, Cat. No. MCA341PE). **(B)** Detection of VCAM1 in aortic sinus by immunohistochemical staining with anti-VCAM1 antibody (Proteintech, Cat. No. 11444-I-AP). **(C,D)** The mRNA level of CD68, VCAM1, ICAM1, E-selectin, and P-selectin in aortas. **(E)** Masson staining of aortic sinuses. Data are shown as mean ± SD. *n* = 4–8. **p < 0.01 and ***p < 0.001 (Student’s *t*-test).

As macrophage infiltration plays an important role in atherosclerosis development (Martens et al., 1998), we proceeded to perform immunohistochemical analysis to see whether there is any change in aortic macrophages. Indeed, increased macrophage content was found in the aortic sinus of *Ldlr*^–/–^*G2a*^–/–^ as compared to *Ldlr*^–/–^ rats (Figure 4A), together with markedly elevated CD68 mRNA level (Figure 4C). In addition, increased staining of the adhesion molecule VCAM1 was also found in the aortic sinus of *Ldlr*^–/–^*G2a*^–/–^ rats, together with increased mRNA levels of VCAM1, ICAM1, and E-selectin, although the VCAM1 change did not reach significance (Figures 4B,D). Moreover, Masson staining revealed more collagen deposition in the vessel wall of *Ldlr*^–/–^*G2a*^–/–^ rats (Figure 4E). Hence the aggravated atherosclerosis in G2A deficient *Ldlr*^–/–^ rats was accompanied by increased macrophage recruitment and vascular fibrosis.

### Enhancement of Macrophage Migration and Apoptosis in the G2A Deficient Atherosclerotic Rats

**FIGURE 5:**
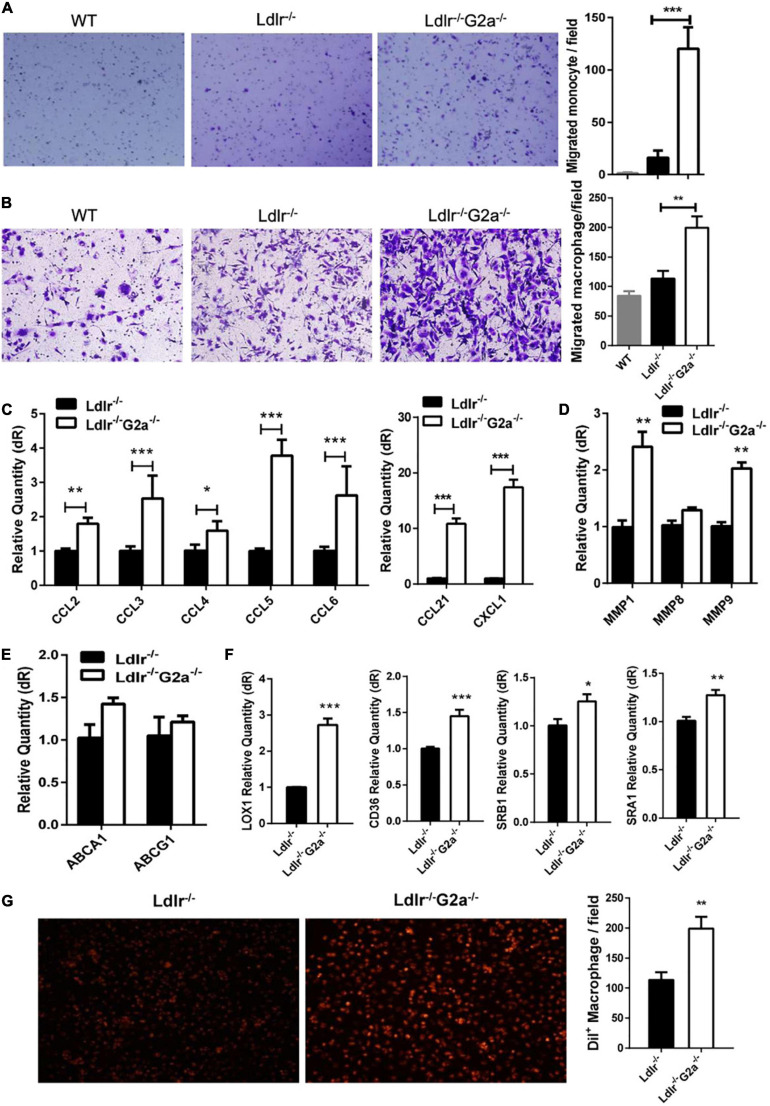
Effect of G2A deficiency on macrophage migration and lipid transportation. **(A,B)** Migration assay for oxLDL stimulated monocytes and macrophages from different genotypes of rats. **(C)** The mRNA level of aortic chemokines. **(D)** Relative expression of MMPs in macrophages. **(E)** Expression of ATP binding cassette transporters in macrophages. **(F)** Expression of different scavenger receptors. **(G)** Dil-oxLDL uptake by *Ldlr*^–/–^ and *Ldlr*^–/–^ *G2a*^–/–^ macrophages. Cells were incubated with 10 μg/ml Dil-oxLDL for 18 h in the dark, then washed and examined under a microscope. Fluorescence was analyzed by Image J. Data are shown as mean ± SD. *n* = 3–9. *p < 0.05, **p < 0.01 and ***p < 0.001.

The above results and G2A single deletion’s effect on macrophages prompted us to analyze functional changes in monocytes and macrophages. Both bone marrow-derived monocytes and macrophages from *Ldlr*^–/–^*G2a*^–/–^ rats exhibited a higher migration ability than those from *Ldlr*^–/–^ rats (Figures 5A,B). Concomitantly, there were consistently and significantly increased aortic chemokines related to monocyte and macrophage recruitment (Figure 5C), indicating increased interactions between these cells and endothelium. The increased macrophage chemotaxis may also attribute to matrix metalloproteinase (MMP) enhancement, as increased expression of MMP1 and MMP9 was found in *Ldlr*^–/–^*G2a*^–/–^ macrophages (Figure 5D). Since the significantly increased CCL2 is related to inflammation, the inflammatory phenotype of macrophages was also examined. After 24 h stimulation by oxLDL, *Ldlr*^–/–^*G2a*^–/–^ macrophages displayed a minor increase in inflammatory IL-1β and reduction in anti-inflammatory IL-10 as compared to *Ldlr*^–/–^ macrophages. In polarization conditions, G2A deficient macrophages showed a significant increase of IL-1β when stimulated by LPS, and decreased TGF and IL-10 when stimulated with IL-4 (Supplementary Figure 7).

The expression of genes encoding ATP-binding cassette transporters, responsible for cholesterol efflux, was unaffected by G2A deficiency as examined by real-time PCR (Figure 5E). However, the expression of scavenger receptors (SRs) including LOX1, CD36, SRA1, and SRB1 were all significantly increased (Figure 5F). Among them, LOX-1 displayed the greatest elevation. SRs mediated oxLDL internalization was also enhanced by G2A deletion (Figure 5G). Intriguingly, the cell number was declined when macrophages were incubated with Dil-oxLDL for 24 h, as compared to 18 h incubation (data not shown), implying possible cell apoptosis.

**FIGURE 6:**
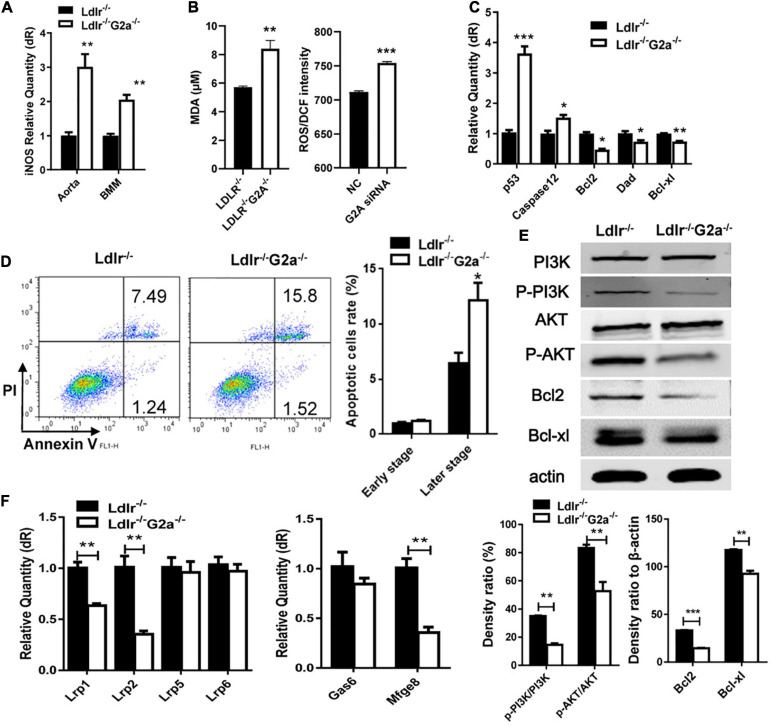
Macrophage apoptosis and efferocytosis in *Ldlr*^–/–^ and *Ldlr*^–/–^ *G2a*^–/–^ rats. **(A)** iNOS expression in aortas and oxLDL-induced macrophages. **(B)** The serum MDA level of the two genotypes, and ROS generation in G2A knockdown HUVEC cells (NC, scrambled siRNA). **(C)** Relative expression of apoptotic genes and survival genes. **(D)** Flow cytometry analysis for Annexin V positive macrophages. **(E)** Immunoblot analysis of proteins of PI3K/AKT pathway in macrophages. The β-actin was used as a control for protein loading. **(F)** Relative expression of efferocytosis related genes. Data are representative of three or more independent experiments and shown as mean ± SD. *p < 0.05, **p < 0.01 and ***p < 0.001 (Student’s *t*-test).

Further examination revealed G2A regulating macrophage apoptosis which is related with oxidative stress. As in WT, the absence of G2A in *Ldlr*^–/–^ rats greatly up-regulated iNOS mRNA expression, both in the aorta and oxLDL stimulated macrophages, suggesting stress associated with increased blood oxLDL and oxLDL uptake. This was confirmed by the elevated serum MDA level in *Ldlr*^–/–^*G2a*^–/–^ rats and ROS generation in G2A knockdown HUVEC cells (Figures 6A,B). As shown in Figure 6C, the expression of apoptotic genes p53 and caspase 12 was enhanced while survival genes (Bcl2, Dad, and Bcl-xl) were reduced in *Ldlr*^–/–^*G2a*^–/–^ macrophages. In addition, flow cytometry analysis (FACSCalibur, Becton Dickinson, United States) manifested significantly elevated Annexin V-positive apoptotic cells (Figure 6D). The result was corroborated by *in situ* TUNEL assay which showed increased corresponding fluorescence in section preparations of *Ldlr*^–/–^*G2a*^–/–^ aortas (Supplementary Figure 8). Apoptotic pathway-related genes were then probed by immunoblotting. There was significantly reduced phosphorylation of PI3 kinase (PI3K) and AKT (Figure 6E and Supplementary Figure 9), as well as a decrease of downstream gene expression including Bcl2 and Bcl-xl. The data suggested possible participation of PI3K/AKT signaling pathway in G2A regulation to macrophage apoptosis. Finally, to examine whether there was any alteration in efferocytosis, the clearance of apoptotic cells, LDLR-related proteins in macrophages were analyzed significantly suppressed expression of LRP1 and LRP2 was detected. Moreover, Gas6 and Mfge8 were inhibited although only the latter reached significance (Figure 6F). Altogether, the above results indicate that G2A deletion induced atherosclerosis aggravation was associated with macrophage changes including increased chemotaxis and apoptosis.

## Discussion

**FIGURE 7:**
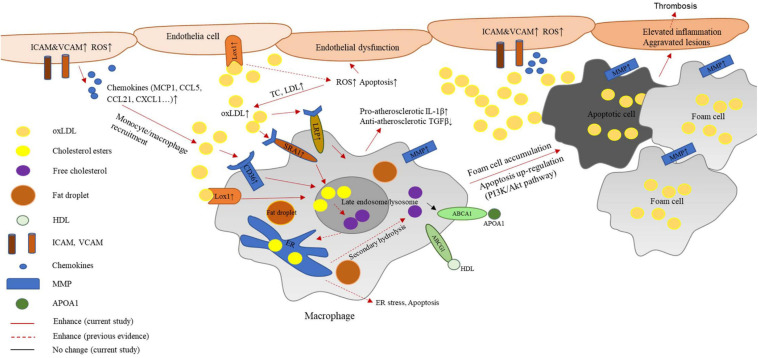
Potential mechanism of G2A deficiency in atherosclerosis development by regulating macrophages. G2A knockout in LDLR deficient background leads to an exaggerated lipid disorder and pro-atherosclerosis phenotype. The latter associates with oxidative stress and endothelial dysfunction. Increased expression of adhesion molecules and chemokines recruits more macrophages to the intima. These macrophages engulf oxidized cholesterol (oxLDL) through scavenger receptors including LOX-1, CD36, and SR-A1. However, ABCA1 and ABCG1 which participate in cholesterol efflux did not change. The disruption of lipid homeostasis in macrophages leads to cholesterol accumulation and formation of foam cells. In addition, inflammatory cytokine production by macrophages shifts them to a more pro-atherosclerosis phenotype. Moreover, PI-3K/AKT signaling pathway was found to participate in the up-regulated apoptosis. All these changes eventually contribute to aggravated aortic lesions.

In the present study, we found a protective effect mediated by G2A in the atherosclerosis model established from LDLR deficient rats. As reported in *Ldlr*^–/–^ mice, rat G2A deficiency did not significantly alter body weight. Very different from mice, though, the absence of G2A in the rat model markedly exacerbated lipid disorder. In addition, rat G2A deficiency promoted oxidative stress and secretion of aortic chemokines and adhesive molecules which attracted macrophages, eventually led to aggravated atherosclerosis. Our data indicate that G2A not only participates in regulating macrophage apoptosis and accumulation but is also involved in lipid metabolism. Intriguingly, the effects of G2A on macrophage and lipid were noticed as early as 8 week-old, before the induction of atherosclerosis. While the major functions of G2A on macrophages were regulating migration and apoptosis as G2A deficiency in both WT and *Ldlr*^–/–^ led to obvious changes in these two phenotypes, G2A deletion aggravated lipid disorder only became eminent in *Ldlr*^–/–^ rats. A scheme summarizing the potential mechanism of G2A deficiency in atherosclerosis development by regulating macrophages has been provided in Figure 7.

Previous reports about G2A regulation to immune cells were mostly based on *in vitro* experiments, stimulated by different lipid activators like LPC and 9-HODE (Radu et al., 2004; Wang et al., 2005; Yang et al., 2005; Frasch et al., 2007). Whether one of these ever-growing activators could become the specific agonist for G2A is debated. Additionally, data from murine models about G2A’s role in atherosclerosis are conflicting. While comparing to mice G2A receptor, the rat homolog bears more similarities with human G2A. Above considerations prompted us to use the rat atherosclerosis model which has been reported to share a more similar phenotype to the human disease (Zhao et al., 2018; Lee et al., 2019). Our investigation in the *Ldlr*^–/–^ rat model found that knockout of G2A exacerbated atherosclerosis. This result agrees with the mice report which described that G2A deletion on *Apoe*^–/–^ or *Ldlr*^–/–^ background contributed to aortic macrophage accumulation and increased atherosclerotic plaques (Bolick et al., 2009), but it is in contrast to the other two in *Ldlr*^–/–^ model which found that mice G2A deficiency attenuated atherosclerosis (Parks et al., 2006, 2009). It is expected that our study may take advantage of the benefits from the rat model and provide new clues for interpreting the role of G2A. Interestingly in this study, the rat G2A was found to strongly modify the lipid profile.

This dramatic impact of G2A on lipid profile in LDLR deficient rats was completely different from mouse studies. Although a few reports suggested G2A may participate in homeostasis of hepatobiliary bile salt and phospholipid that is important for gallstone formation (Johnson et al., 2008), analysis of blood lipid profiles usually presented no significant change in G2A deficient atherosclerotic mice (Parks et al., 2005; Bolick et al., 2009). Some studies observed HDL elevation only after extended treatment with Western diet or high-cholesterol diet in *Ldlr*^–/–^ mice (Parks et al., 2006, 2009). It is well-known that dyslipidemia could lead to inflammation. Here the intriguing result that G2A deficiency in *Ldlr*^–/–^ rats induced a marked elevation of dyslipidemia implied that aberrant lipid metabolism may cross-regulate with macrophages and endothelium in atherosclerosis development. Especially, the enhanced inflammatory phenotype in macrophages might attribute to hypercholesteremia induced by G2A depletion, as obvious inflammatory macrophages were only observed in *Ldlr*^–/–^*G2a*^–/–^ rats along with the elevated lipid disorder, not in single G2A knockouts.

As a stress sensor that monitors oxidative state (Obinata and Izumi, 2009), G2A deletion in rats led to arterial stress with increased ROS and oxLDL levels, thus contributed to more macrophage accumulation. Here macrophages may promote atherosclerosis progression through different aspects including migration and apoptosis. The enhanced monocyte and macrophage migration due to G2A deletion brought about elevated macrophage recruitment to the vessel wall, as previously reported in *Ldlr*^–/–^*G2a*^–/–^ mice (Bolick et al., 2007). G2A knockdown had also been reported to increase macrophages in mouse neurons and prolong inflammatory hyperalgesia (Su et al., 2018). However, some mice reports stated no change in macrophage chemotaxis by G2A deletion in *Ldlr* knockouts (Parks et al., 2005). In addition to elevated aortic chemokines and adhesion molecules, the increased macrophage migration in our study may also be attributable to MMP1 and MMP9 enhancement. The oxLDL up-regulates MMPs production from macrophages (Chase et al., 2002). Among them, MMP9 is well-known for its induction of cell migration including macrophages and vascular smooth muscle cells, as well as its promotion to macrophage invasion through the intima (Newby, 2007; Yang et al., 2015; Webb et al., 2017). Recently a study found that both MMP1 and MMP9 were closely linked with aldosterone-induced macrophage migration and infiltration in the myocardium (Liao et al., 2020). MMP9 is also known to associate with collagen accumulation in hypertensive arterial walls (Wang et al., 2015).

Apart from migration, macrophage apoptosis also participates in G2A regulating atherosclerosis, which seems related to PI-3K/Akt pathway. However, there may be other pathways or components involved. As shown in Figure 7, G2A deficiency-induced elevation in LDL and ox-LDL intake leads to lipid accumulation in macrophages, which may cause activation of the ER stress-mediated pathway and promote apoptosis (Chistiakov et al., 2016; Sukhorukov et al., 2020). The increased accumulation of apoptotic cells further enhanced lesion development and macrophage content. Here decreased efferocytosis may also contribute to the accumulating process, as previous reports suggested G2A mediate efferocytosis following tissue injury (Kabarowski, 2009; Frasch et al., 2011; Kern et al., 2018). Since macrophage death is often observed in rupture-prone human and mice plaques (Johnson and Jackson, 2001), increased macrophage apoptosis together with increased MMPs is frequently linked with plaque instability. Moreover, formerly MMP1 was reported to enhance the senescence of endothelial cells via p53 activation (Struewing et al., 2009). In this study we found both increased MMP1 and p53 expression in G2A deficient macrophages, implying G2A deletion may exert an effect on macrophage senescence, which might eventually lead to autophagy. Moreover, one of the downstream genes of the PI3K/Akt pathway, mTOR, inhibits autophagy (Glick et al., 2010), so the inhibited PI3K/Akt pathway by G2A deletion implies possible autophagy involvement. Considering the current study and previous rat atherosclerotic investigations (Wei et al., 2015; Sithu et al., 2017; Zhao et al., 2018), we suspect rats may more accurately model macrophage functions involved in atherosclerosis.

While plaque formation is a consequence of immune cell migration, the initiation of atherosclerosis is thought to relate to endothelial dysfunction. Although more work needs to be done, we did find that G2A in the endothelium may play an important role in atherosclerosis regulation. In addition to the elevated expression of adhesion molecules in G2A deficient aortic sinus, aortic chemokines were also markedly enhanced. These led to macrophage recruitment, indicating promoted endothelial-monocyte/macrophage interaction. We also found that G2A knockdown in HUVEC cells promoted ROS generation. These data support endothelial G2A’s anti-atherosclerosis effect (Bolick et al., 2007), and may partially explain why macrophage changes were noticed even before the induction of atherosclerosis.

In the future, more solid investigation is essential to elucidate functions of endothelial G2A. For macrophage, a rat model with specific G2A deficiency in this cell type might be more useful to determine the exact effect of macrophage G2A on atherosclerosis. Other immune cells, such as T cells may also play their parts in G2A mediated atherosclerosis development (Radu et al., 2004). In addition, it is not certain how much of these rat data could be translated to human disease. Despite these limitations, the present research offers a new angle to look at the function of G2A in atherosclerosis initiation and development.

In summary, this study enriched our understanding of G2A in atherosclerosis development. The rat data demonstrate G2A regulating atherosclerosis by modifying lipid disorder and macrophage functions. Increased atherosclerosis in *Ldlr*^–/–^*G2a*^–/–^ rats may partially attribute to multiple alterations in macrophages including up-regulated migration and apoptosis. More importantly, the marked regulation of lipid disorder presents a new dimension about the role of G2A. It is possible that by modulating G2A signaling in both lipid metabolism and macrophages, we may find a way to develop more powerful therapies for atherosclerosis.

## Data Availability Statement

The original contributions presented in the study are included in the article/Supplementary Material, further inquiries can be directed to the corresponding author/s.

## Ethics Statement

The animal study was reviewed and approved by the Animal Ethics Committee of East China Normal University.

## Author Contributions

HC, BZ, ML, and DL conceived the experiments. XC, RX, YT, MW, YS, YXu, YY, YZ, LX, and YXi conducted the experiments. XC, RX, YT, MW, YS, YXu, and DL analyzed the results. XC, RX, and YXi drafted the work. HC, BZ, and ML wrote and revised the manuscript. All authors reviewed and approved the manuscript.

## Conflict of Interest

The authors declare that the research was conducted in the absence of any commercial or financial relationships that could be construed as a potential conflict of interest.

## Publisher’s Note

All claims expressed in this article are solely those of the authors and do not necessarily represent those of their affiliated organizations, or those of the publisher, the editors and the reviewers. Any product that may be evaluated in this article, or claim that may be made by its manufacturer, is not guaranteed or endorsed by the publisher.

## Abbreviations

LDLR: low-density lipoprotein receptor
LRP: LDLR-related protein
oxLDL: oxidized low-density lipoprotein
LPC: lysophosphatidylcholine
9-HODE: 9-hydroxyoctadecadienoic acid
ROS: reactive oxygen species
WT: wild type
MMP: matrix metalloproteinase.
