# Supplementary material for: The G2A Receptor Deficiency Aggravates Atherosclerosis in Rats by Regulating Macrophages and Lipid Metabolism
Source: Front Physiol. 2021 Jul 26;12:659211. doi: 10.3389/fphys.2021.659211 (PMC8351205; doi:10.3389/fphys.2021.659211)
Supplement: Supplementary file 1 [file Data_Sheet_1.PDF]

Supplementary Materials

Supplementary Figures and Figure Legends

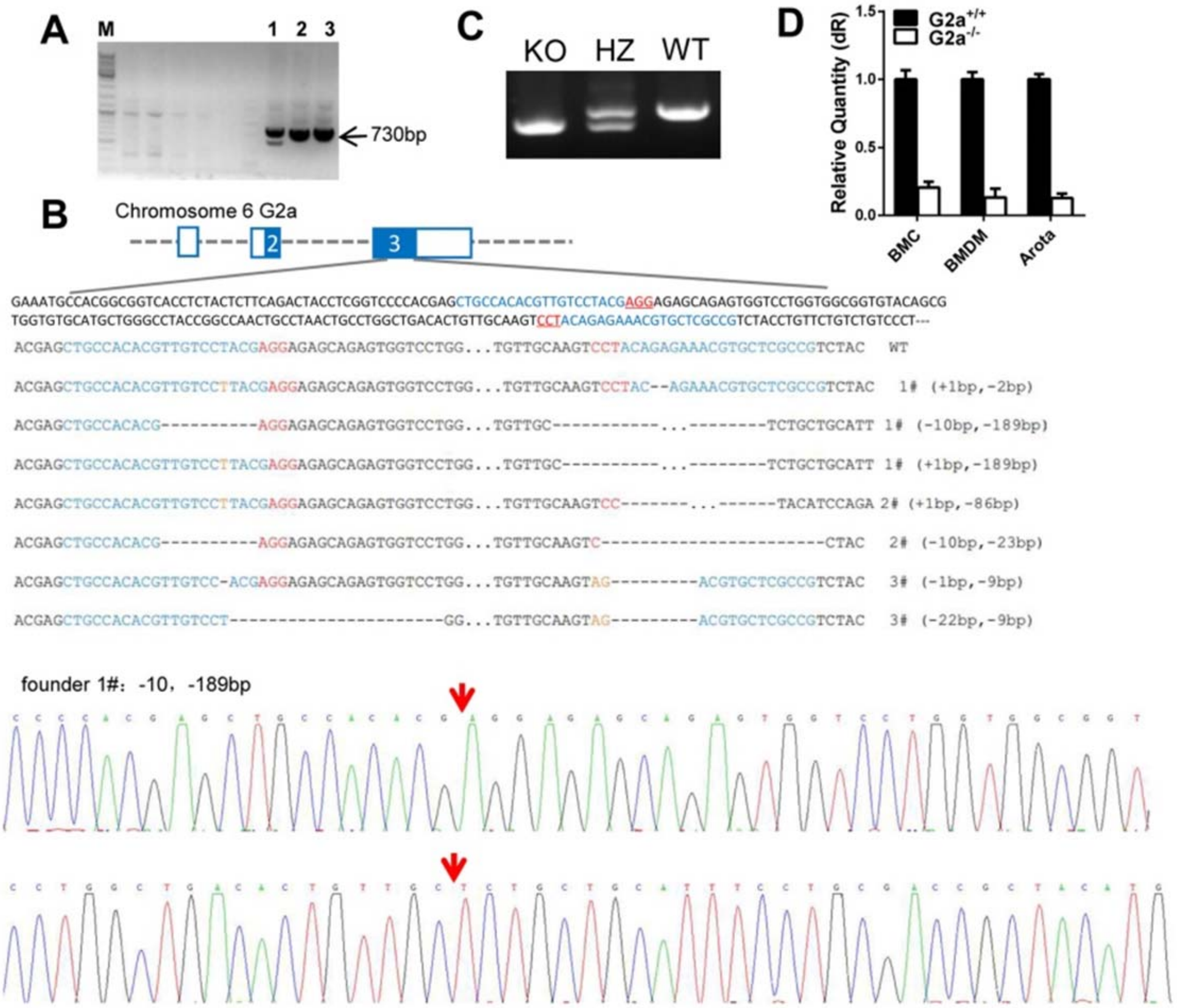

Supplementary Figure S1 Generation of the *G2a* knockout rat by CRISPR/Cas9 system. (A). Target loci of *G2a* were amplified using genomic DNA templates from founders. M: DNA molecular weight marker generuler 0331; 1-3: Founder rats generated by microinjection. (B). Top: PCR products of the targeted fragment in the *G2a* in rats were sequenced. The protospacer adjacent motif (PAM) sequence was underlined and highlighted in red; the targeting sites were blue; the insertions were orange; insertions (+) or deletions (-) were shown to the right of each allele. Bottom: DNA sequencing map of founder 1. (C). Typical genotyping result. (D). *G2a* expression in different tissues from WT and *G2a* knockout rats. BMC, bone marrow cells. BMDM, bone marrow derived cells.

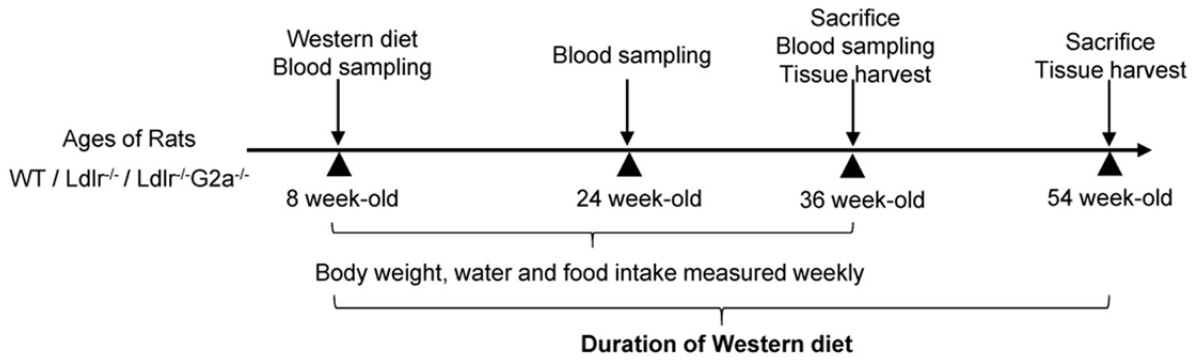

Supplementary Figure S2 The scheme depicting the time protocol of rat treatments and time points of measuring different parameters.

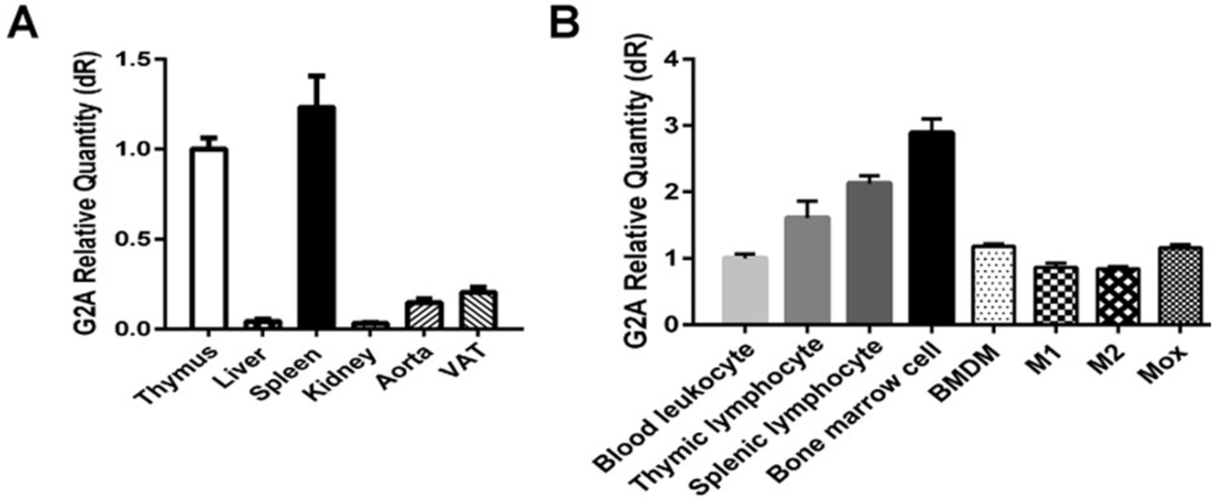

Supplementary Figure S3 Rat G2A expression in atherosclerosis related tissues and cells. (A). Real-time PCR analysis performed in triplicate on total RNA from different tissues. (B). G2A expression by various immune cells. VAT, visceral adipose tissue. BMDM, bone marrow derived macrophages. M1 & M2, macrophages polarized with LPS and IL-4 respectively. Mox, oxLDL induced macrophages. Data are shown as mean ± SD.

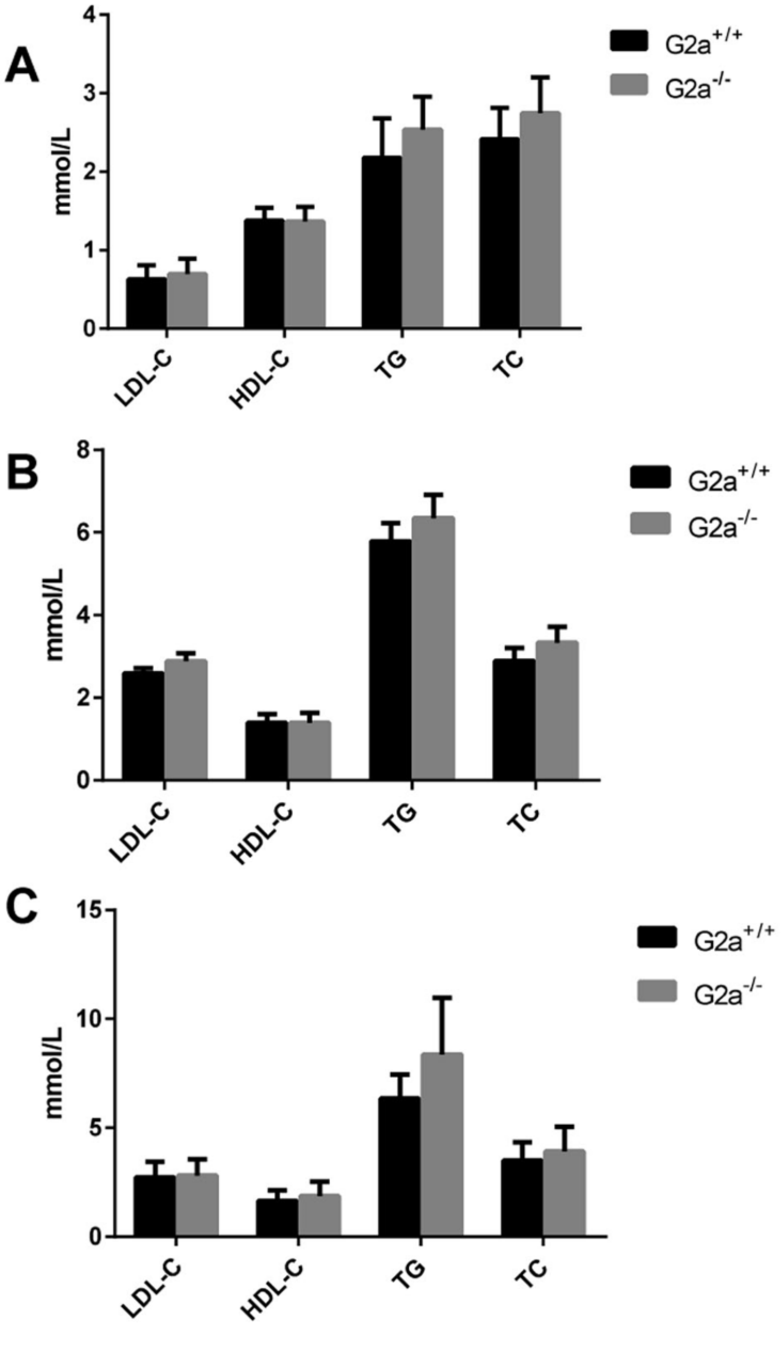

Supplementary Figure S4 Effect of G2A deletion on lipid metabolism of WT rats fed by normal or Western diet. (A&B). Lipid profiles of normal diet-fed rats at 8 and 36 week-old (n = 5 & 12 respectively). (C). Lipid profiles of Western diet-fed rats at 36 week-old (n = 7). Data are shown as mean ± SD.

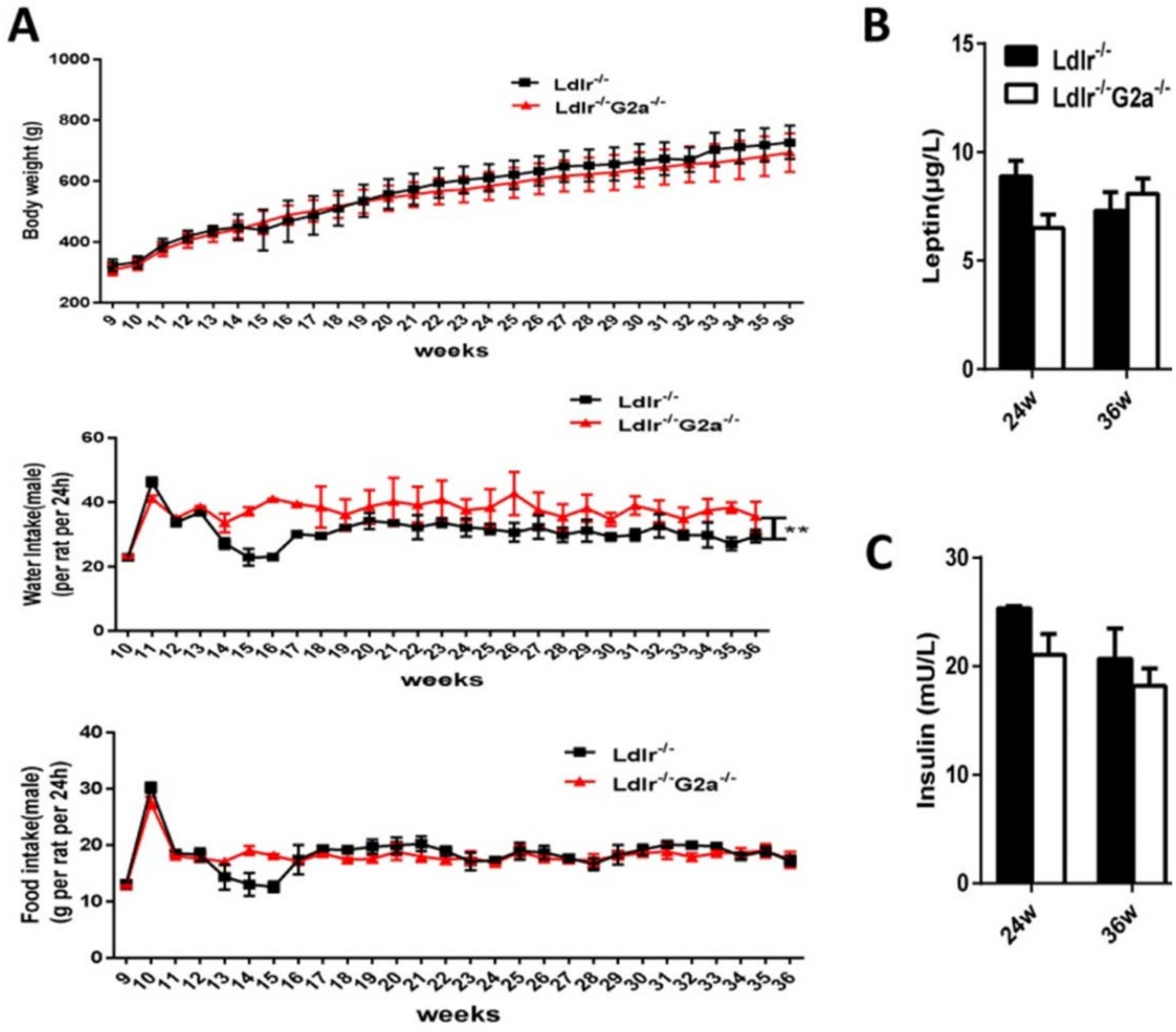

Supplementary Figure S5 Effect of G2A deficiency on body weight, water intake, food intake, leptin and insulin. (A). Body weight, water and food intake of *Ldlr*$^{-/-}$ *and Ldlr*$^{-/-}$*G2a*$^{-/-}$ rats. (B). Leptin levels of 24 and 36 week-old rats. (C). Insulin levels of 24 and 36 week-old rats. Data are shown as mean ± SD. n = 4-8. **p < 0.01.

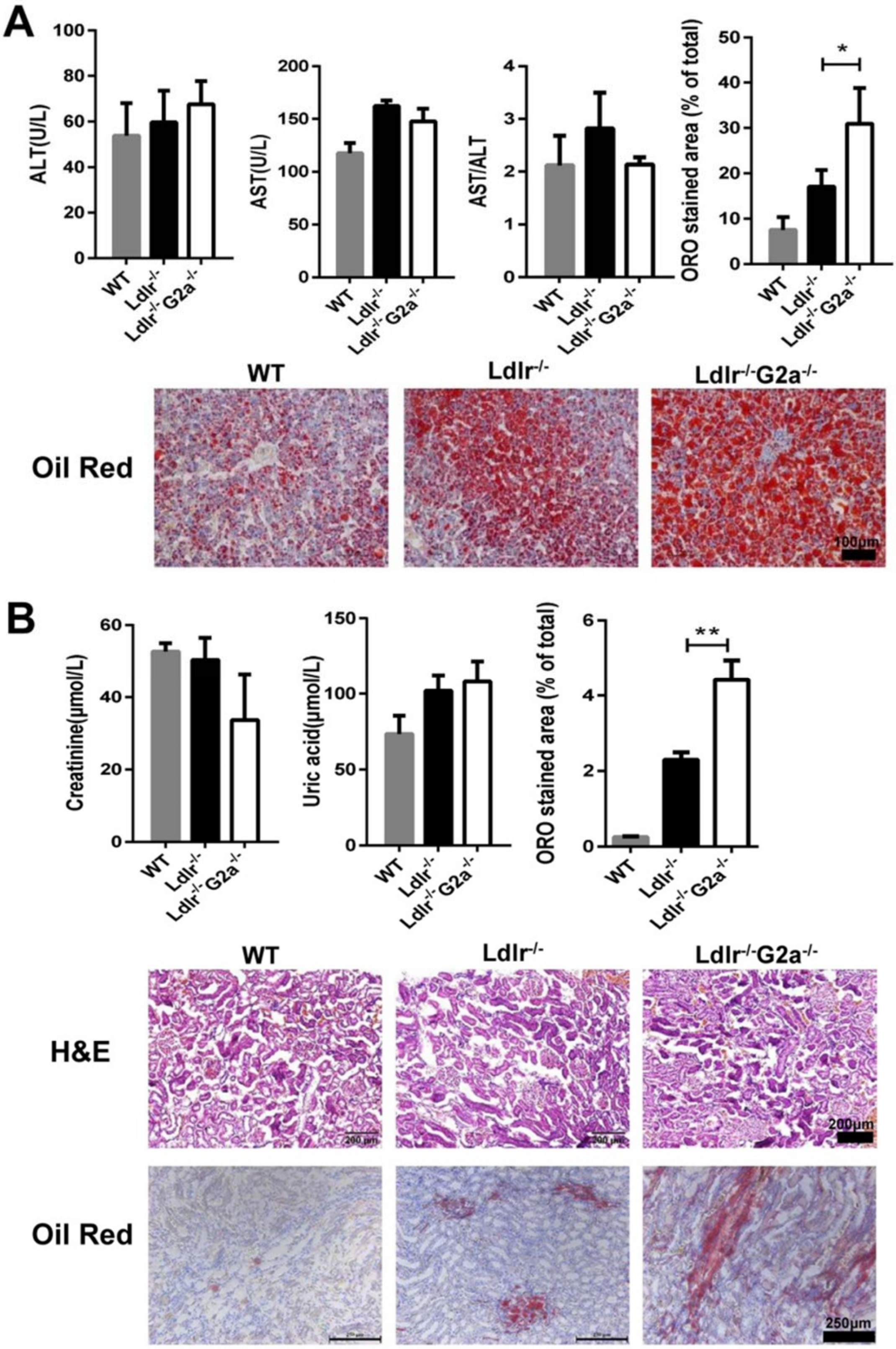

Supplementary Figure S6 Effect of G2A deficiency on liver and kidney. (A). AST, ALT and lipid deposition in livers of WT, *Ldlr*$^{-/-}$ *and Ldlr*$^{-/-}$*G2a*$^{-/-}$ rats. (B). Kidney functional indexes and lipid deposition of the three genotypes at 24 week-old. Data are shown as mean ± SD. n = 4-8. *p < 0.05, **p < 0.01 vs. *Ldlr*$^{-/-}$ rats.

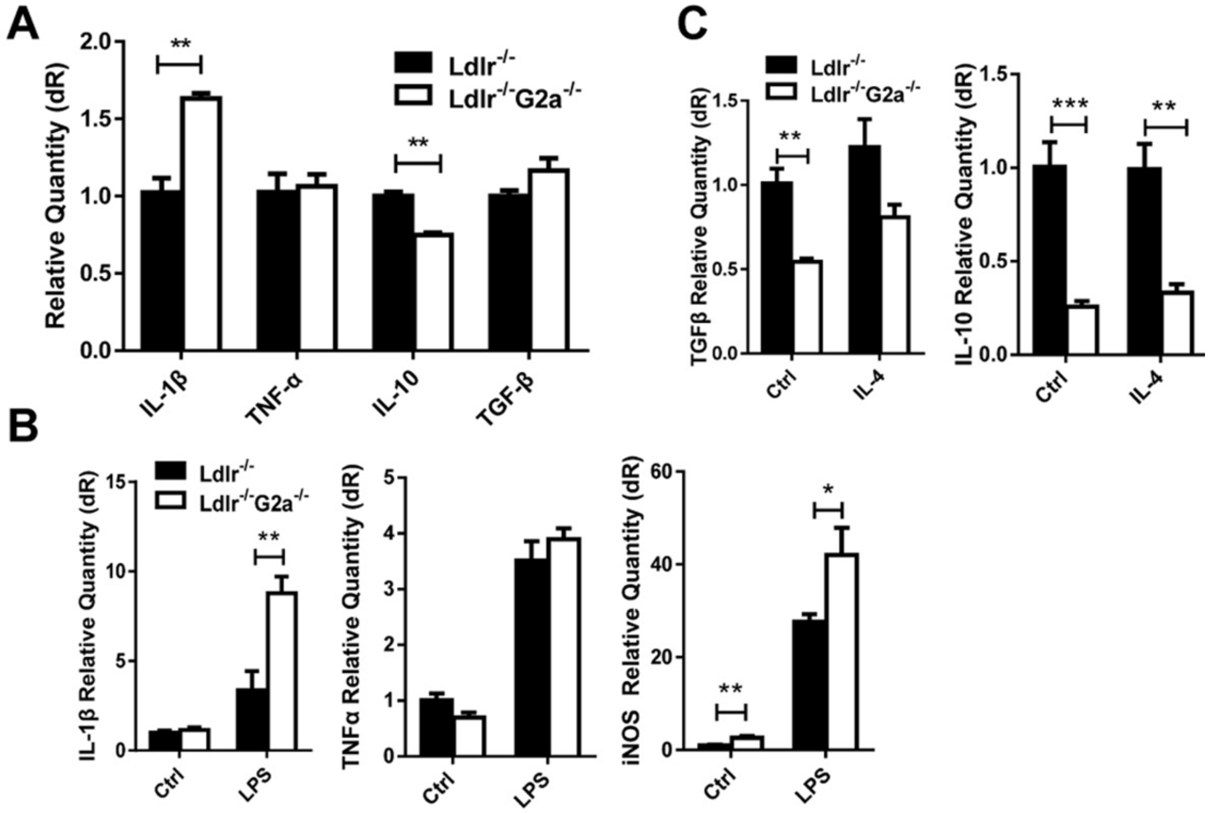

Supplementary Figure S7 Effect of G2A deficiency on cytokine profile of macrophages. (A). Real-time PCR analysis of cytokines produced by macrophages after 24 h stimulation of oxLDL. (B). The mRNA level of inflammatory cytokines in LPS stimulated macrophages. (C). Anti-inflammatory cytokines produced by macrophages with IL-4 stimulation. Macrophages were obtained from 36 week-old *Ldlr*$^{-/-}$ and *Ldlr*$^{-/-}$*G2a*$^{-/-}$ rats. Data are representative of three independent experiments and shown as mean ± SD. *p < 0.05, **p < 0.01 and ***p < 0.001 (Student's t-test).

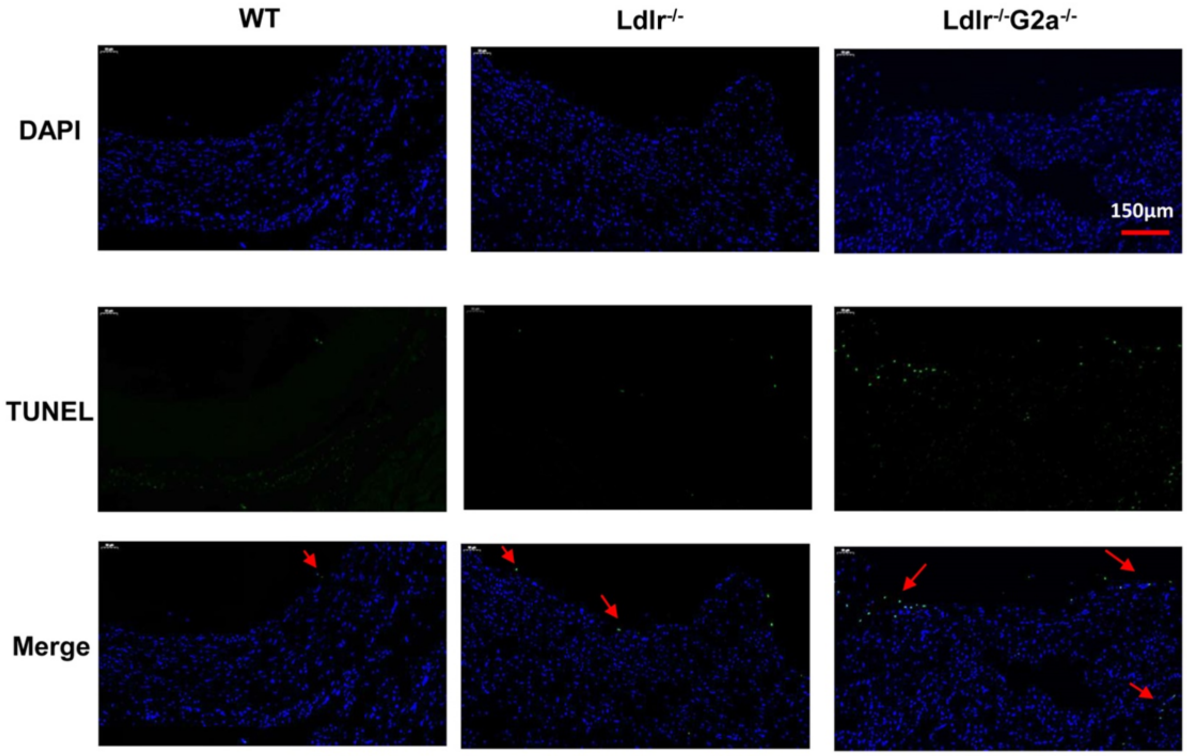

Supplementary Figure S8 Lesional apoptosis analysis by TUNEL assay in 54 week-old rats. The analysis was performed in aortic sinus of the indicated genotypes by the In situ Cell Death Detection (TUNEL) kit (Roche, Cat. NO. 11684795910) according to the manufacture's instruction. Images were captured using a microscope scanner-Pannoramic MIDI (3D HISTECH). Blue for nuclei and green for apoptotic cells.

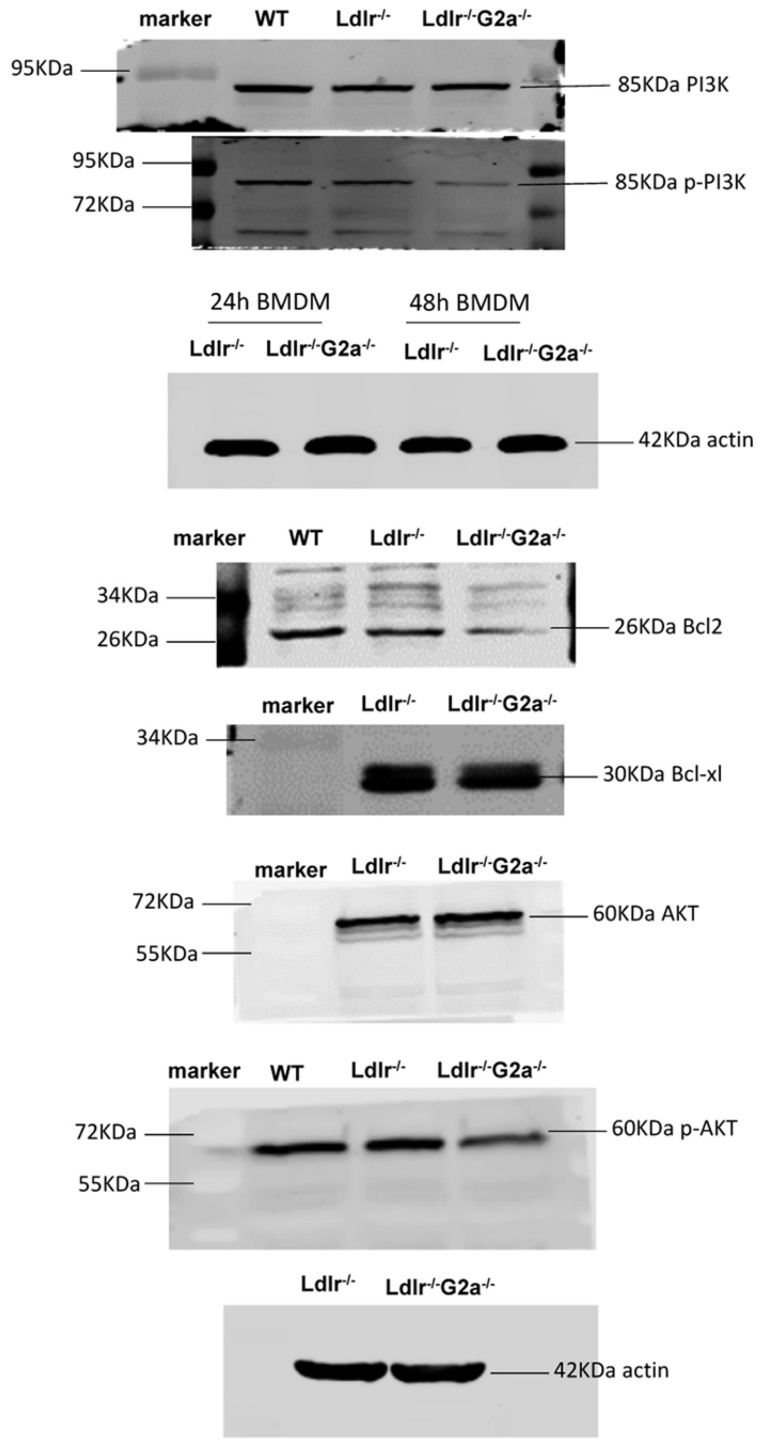

Supplementary Figure S9 The original Western blots of proteins in PI-3K/Akt pathway. All blots were from bone marrow derived macrophage (BMDM) stimulated by 10 μg/ml oxLDL for 24 h, except half of the β-actin blot labeled with 48h BMDM (BMDM stimulated by oxLDL for 48 h).

Supplementary Table 1 Primers for Quantitative Real-time PCR

Genes	Forward primers (5'-3')	Reverse primers (5'-3')
IL-1β	TAGCAGCTTTCGACAGTGAGG	CTCCACGGGCAAGACATAGG
β-actin	CGTTGACATCCGTAAAGA	AGCCACCAATCCACACAG
IL-10	ACGCTGTCATCGATTTCTCCC	ATAGGTCGGGCCCTCTTTCT
ICAM-1	CTTTGCCCTGGTCCTCCAAT	GTTAGTCTCCAACCCCAGGC
VCAM-1	TCTACAGCTCCTCTCGGGAA	AACGGAATCCCCAACCTGTG
TNFα	ATGGGCTCCCTCTCATCAGT	GCTTGGTGGTTTGCTACGAC
TGFβ	GACCGCAACAACGCAATCTA	TTCCGTCTCCTTGGTTCAGC
LOX-1	TGAACTTCGTCTTACAAGCA	CCCCTGGTCCTAAAGAATTG
CD36	GAGGTCCTTACACATACAGAGTTCGTT	ACAGACAGTGAAGGCTCAAAGATG
NOS2	CACAGTGTCGCTGGTTTGAA	TCTCCGTGGGGCTTGTAGTT
SRA1	AGAAGATGCTGGGGTCACTTG	TTTAGTAGAGCAGGGCAACAA
SRB1	TCGAACAGAGCGGGATGATG	TTGGCTTCTTGCAGTACCGT
ABCA1	CTGGTCAAGAAAGATGTGGA	GAGACATCTTGAGTCAGCG
ABCG1	CTGTCTGATGGCCGCTTTCT	TTGTATCCTTTCTTCCTCCACCAG
P53	CCCACAGAATGACTTGCCCT	TTTAGGCTGCTCCTATCGGC
CCL2	CTGTGCTGACCCCAATAAGGA	ACAGAAGTGCTTGAGGTGGT
CCL3	AGCCAGCTGTGGTATTTCTGAC	AGTGGCTCTTCCTGTCTTGAG
CCL4	AGCCAGCTGTGGTATTTCTGAC	AGTGGCTCTTCCTGTCTTGAG
CCL5	TCCCCATATGGCTCGGACA	ATCCCCAGCTGGTTAGGACT
CCL6	TATCCTTGTGGCCGTCCTTG	GGTGACAAAGATGATGCCTGG
CXCL1	GATGCTAAAGGGTGTCCCCAA	ACGACCATCGATGAAACGCA
CD68	AATGGTTCCCAGCCATGTGT	AATGTCCACTGTGCTGCTTG
BCL2	TGGCCTTCTTTGAGTTCGGT	GTTCCACAAAGGCATCCCAGC
Caspase12	TCCGAAAGGTTCAATACTCATTTG	ACATCTGGGGATCTTGGTATTTTCT
Gas6	CCTCAACTACACCCGGACATC	GCCAGGGCAACATTCTCAAC
Mfge8	ACTGTGAACTCGGCTGTTCC	TCTTCCGCAGAAAGTCCACC
BCL-xL	AGCAGTCAGCCAGAACCCTA	CCCGGTTGCTCTGAGACATT
Dad	AGCTTCATCTTAGCGGTTTGC	ACGGTAAGGAAATGGGAGCG
CD68	TGTGTCCTTCCCACAAGCAG	GAGAAGCATGGCCCGAAGT
MMP1	TCAGCATGCTTAGCCTTCCT	AGGTATTTCCAGACTGTTTCCAC
MMP8	CAAAGGAAGGCACGAGAGGT	CGTAGGTAATTCTCAGCAGTTTTCA
MMP9	TCTGCCTGCACCACTAAAGG	CAGGCTGTACCCTTGGTCTG
CCL21	ACATTGTCCGAGGCTACAGG	AGTTTTGTTTCCCTGGGGCT
LRP1	GGCCGATGCATCCCTATCTC	TCATTGTCGGCAGGCTCAGG
LRP5	ACTTGTGGTGAGCCTCCTACC	GTTAGCTTCATCCGAGCCATC
LRP6	GCTCGGAGTCTCAGTTCCAG	TGGCTCCTCAGTTGGATAGCA
P-selectin	TTCCGGTCCCAAGTAAAGCC	CAGTGTCCAGTAGCCAAGCA
E-selectin	GAAGAGACCCAGCAAGCCAT	ACCCATACCCACCATCACACC
